# Development of Compounded Surfactant Foam and Its Application in Emergency Control of Piping in Dikes

**DOI:** 10.3390/molecules30122583

**Published:** 2025-06-13

**Authors:** Jiakun Gong, Zuopeng Pang, Yuan Wang, Jie Ren, Tian Qi, Adam Bezuijen

**Affiliations:** 1 College of Mechanics and Engineering Science, Hohai University, Nanjing 210098, China; jiakungong@hhu.edu.cn (J.G.);; 2 College of Water Conservancy & Hydropower Engineering, Hohai University, Nanjing 210098, China; 3 Department of Civil Engineering, Ghent University, 9000 Ghent, Belgium

**Keywords:** dike, piping, anion–cation surfactant, synergistic effect, foam

## Abstract

Piping is a severe threat to dikes, which can lead to dike failure, and cause significant economic and human casualties. However, conventional measures necessitate substantial labor and material resources. A novel foam-based method for the rapid mitigation of piping was proposed to enhance piping emergency control efficiency, which demonstrates significant application potential. This study aims to develop a novel foam formulation and evaluate its performance in controlling piping in dikes. Through a combination of foam static-property characterization experiment and foam plugging capacity assessment experiment, a compounded anionic–cationic surfactant composed of sodium dodecyl sulfate (SDS) and cetyltrimethylammonium bromide (CTAB) is optimized. The formulation, at a 9:1 mass ratio and 1.5% total concentration, exhibits superior foam stability and plugging performance. An experiment on the ability of the foam to restrain piping demonstrated that, compared to single-component SDS foam, the compounded SDS-CTAB foam increased the critical hydraulic gradient for piping from 2.35 to 2.70, a 15% improvement. It also reduces the extent of piping channel development under equivalent hydraulic conditions. The foam storage area exhibits enhanced scour resistance and better preservation under prolonged water flow. Mechanistically, the SDS-CTAB foam benefits from synergistic hydrophobic interactions, electrostatic attraction, and hydrogen bonding between surfactant molecules, which enhance foam stability.

## 1. Introduction

China is one of the countries that suffer severely from flood disasters, and the situation is becoming even worse due to the extreme rainfall caused by climate change [1]. An example of this is the flood even that occurred in the whole Yangtze River Basin in 2020, which affected over 34 million people and resulted in a loss of CNY 130 billion in economic terms [2]. In order to reduce the human and economic cost of flooding, more than 410,000 km dikes have been built in recent decades, which form the foundation of the flood control system of China [3]. However, due to complications in the construction process and quality defects, the dikes still face a serious threat of piping. Piping is a major cause of seepage failures in hydraulic structures like dikes [4]. Reports show that over 60% of the dike failures during the 1998 Yangtze River flood were caused by piping [5]. In 2024, piping occurred in the dike of Tuanzhou Bank, which is located along the Dongting Lake. This event ultimately caused the dike to breach. Therefore, it is crucial to deal with piping quickly and effectively once it happens.

The current emergency response to piping incidents still predominantly relies on conventional methods. Upon identification of a piping orifice, the primary interventions involve constructing a reverse-filter well to mitigate particle loss through filtration suppression, or alternatively, deploying weighted covers and implementing water storage counter-pressure to reduce the hydraulic gradient [6,7]. These measures ultimately decrease the likelihood of piping occurrence within the dike foundation.

The conventional measures for combating piping risks during flood seasons necessitate substantial labor and material resources, which significantly compromises emergency response efficiency. Wang et al. [8,9] proposed a novel foam-based method for rapid mitigation of piping.

**Figure 1:**
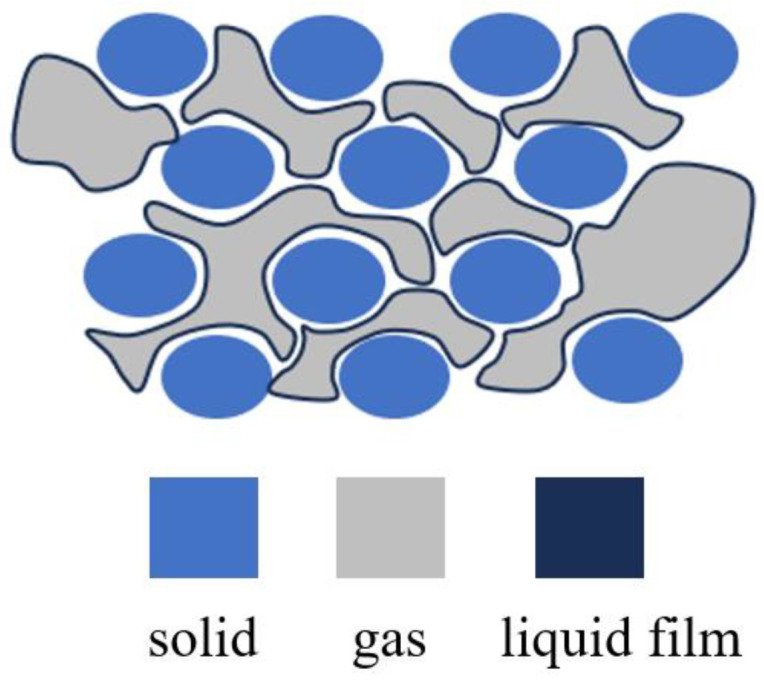
Schematic of foam in porous media.

In porous media, foam is a dispersed system formed through the dispersion of gas in liquid [10]. In this system, the liquid serves as the continuous phase, while the gas is the discontinuous phase separated by liquid films (Figure 1). The foam bubbles in porous media are approximately the same size as the pores, but much larger than the throat size. Foam is widely used in a variety of fields, such as enhanced oil recovery [11,12,13], aquifer remediation [14,15,16], CO_2_ sequestration [17,18,19], and EPB shield tunneling [20], due to its unique rheological properties in porous media. One special property of foam is that it can serve as a blocking agent. When foam bubbles pass through pore throats, they deform under capillary action, creating additional flow resistance (known as the Jamin effect), as illustrated in Figure 1. This significantly increases the overall flow resistance of the fluid, thereby achieving fluid-blocking functionality [21]. This allows it to play a crucial role in inhibiting the formation and expansion of seepage channels.

Wang et al. found that foam can significantly reduce soil permeability, inhibit particle migration within the soil, extend the seepage path of water, increase the critical hydraulic gradient for initiating seepage failure, and enhance flow resistance, thereby effectively increasing the critical hydraulic gradient for piping and suppressing the development of piping [8,9]. Foam exhibits considerable potential for emergency control of piping in dikes. However, current research on this novel approach is still in the preliminary stage, with several key challenges remaining to be addressed. One challenge is that of enhancing the stability and other properties of foam, thereby improving its ability to mitigate the development of piping.

Previous studies have shown that single-surfactant foams, such as those prepared with alpha-olefin sulfonate (AOS), often exhibit limited stability under high hydraulic gradients, leading to rapid drainage and film rupture. For instance, in a previous work [9], AOS-based foam was injected 15 cm upstream of the piping outlet for 60 min, resulting in a critical hydraulic gradient of only 1.84. This indicates insufficient plugging effectiveness under sustained hydraulic stress, highlighting the need for more stable and robust foam formulations for seepage control applications.

Under high hydraulic gradients, foam is subjected to destabilizing forces that can lead to structural failure. The primary mechanisms of foam destabilization include liquid drainage [22], film thinning, bubble coalescence, and gas diffusion (coarsening) [23]. Hydraulic shear and elongational stresses can accelerate these processes [24], leading to rupture of foam lamellae and collapse of the foam structure. These effects reduce the foam’s ability to block seepage paths and resist soil erosion. Therefore, improving foam stability under hydraulic stress is critical for its effective application in dike piping control.

The methods for enhancing the properties of foam mainly include nanoparticle reinforcement [25,26,27], polymer stabilization [28,29,30], and the synergistic effect of surfactant compounding [31,32,33].

Meng et al. found that hydrophobic silica nanoparticles can form stable particulate layers at foam lamellae, significantly enhancing foam stability [34]. The mixture of polymer and surfactant demonstrates exceptional performance in enhancing foam shear resistance and improving viscoelastic properties [35]. Surfactants serve as the predominant factor in determining the characteristics of foam [36]. According to the nature of their hydrophilic headgroups, surfactants can be classified into four major categories: anionic, cationic, nonionic, and zwitterionic surfactants [37]. Binary surfactant mixtures typically consist of nonionic/nonionic, anionic/nonionic, and cationic/nonionic surfactants [38]. The synergy between these surfactants depends on the shape, size, and chemistry of their molecules, such as hydrocarbon chain lengths, branches, charge differences, hydrogen bonds, and so on [39,40,41,42,43].

Among various binary surfactant systems, anionic–cationic combinations (such as SDS and CTAB) exhibit particularly strong synergistic effects [41]. These arise primarily from electrostatic attractions between oppositely charged headgroups, which promote the formation of compact, stable mixed micelles and bilayer structures at the gas–liquid interface. This close molecular packing enhances interfacial surface coverage, reduces surface tension more efficiently, and significantly improves the elasticity and thickness of foam films [44]. In addition, the formation of ion pairs can effectively suppress bubble coalescence and liquid drainage, both of which are critical under high hydraulic gradients. As a result, anionic–cationic surfactant systems tend to produce stronger, more resilient foams that better withstand hydraulic shear forces and prolong foam persistence in complex porous media environments.

In this study, we address the specific requirements for employing foam in emergency mitigation of piping in dikes. First, a surfactant screening method is established, which comprehensively considers the performance of foam and its effectiveness in suppressing the development of piping. Then, based on the synergistic effect of surfactant compounding, a new surfactant formulation suitable for the emergency treatment of piping is developed. Finally, the efficacy of the novel foam in suppressing the initiation and progression of piping is validated through comparative experiments, together with mechanical analysis.

## 2. Results and Discussion

### 2.1. Development of Compounded Surfactant Formulation

In this section, we develop a new formulation of compounded surfactant for enhancing the capability of foam to restrain piping. First, 14 individual surfactants are screened using the Waring–Blender method to select the best-performing single-component surfactants. Then, the selected surfactants are compounded at different ratios, and four preferred compounded formulations are chosen by applying the Waring–Blender method. Finally, the plugging performance of the foam generated by the compounded surfactants is evaluated to determine the optimal surfactant formulation.

#### 2.1.1. Initial Selection of Single-Component Surfactant

By analyzing widely used surfactants with excellent performance, four categories—anionic, amphoteric, nonionic, and cationic surfactants—are selected, comprising a total of 14 surfactants for initial selection. The key parameters of single-component surfactant foam—foam volume (*V*_0_), drainage half-life (*t*_1/2_), and foam comprehensive value (*FCI*)—are listed in Table 1.

As shown in Table 1, among the anionic surfactants, sodium alpha olefin sulfonate (AOS) and sodium dodecyl sulfate (SDS) exhibit high foam comprehensive values (*FCI*) at all concentrations. Furthermore, when the surfactant concentration exceeds 0.3%, SDS demonstrates superior foam performance to AOS. This is primarily due to the presence of long hydrophobic chains in both SDS and AOS [45], which effectively reduce the surface tension of water, resulting in more stable foam. Considering both foaming ability and foam stability, SDS is selected as the optimal anionic surfactant for this study.

The foam comprehensive values of zwitterionic surfactants fluctuate significantly across different concentrations. Lauryl propyl betaine (LAB-35) and lauramidopropyl hydroxy sulfobetaine (LHSB) show poor foam performance at low concentrations, with significant fluctuations. In contrast, cocoamidopropyl betaine (CAB-35) exhibits consistently high foam comprehensive values across the surfactant concentration range of 0.1% to 0.9%, peaking at 0.9%, demonstrating excellent foam property. Therefore, CAB-35 is selected as the optimal zwitterionic surfactant for this study.

Nonionic surfactants exhibit relatively good foam stability, but their foaming ability is poor. Overall, the foam comprehensive value of nonionic surfactants increases with surfactant concentration, showing a trend where higher concentrations result in better foam performance. Among them, alkyl glycoside (APG1214) exhibits the highest foam comprehensive value at all concentrations. Therefore, APG1214 is selected as the optimal nonionic surfactant for this study.

Due to the limited variety of cationic surfactants, this study examines only two cationic surfactants. The foam comprehensive value of cetyltrimethylammonium bromide (CTAB) is highest at 0.9%, significantly outperforming dodecyl trimethyl ammonium chloride (DTAC), demonstrating excellent foam performance. Therefore, CTAB is selected as the optimal cationic surfactant for this study.

In summary, through the screening and evaluation of different surfactants, SDS, CAB-35, APG1214, and CTAB are chosen as the preferred single-component surfactants from the anionic, zwitterionic, nonionic, and cationic categories, respectively. Based on these findings, further research on compounded surfactant formulation is conducted.

#### 2.1.2. Selection of Compounded Surfactant Formulation

As discussed above, the surfactant sodium dodecyl sulfate (SDS) exhibits good foaming ability and foam stability at various concentrations. In addition, previous work shows that the surfactant SDS demonstrates excellent synergistic and interfacial effects when compounded with other surfactants [46,47]. Therefore, in this study, SDS is selected as the primary surfactant, and compounded with the other selected surfactants. The concentration of each surfactant is set to 1%, as this concentration ensures relatively stable properties and good foam performance.

- Synergistic Effects of Anionic–Cationic Surfactant Mixtures

**Figure 2:**
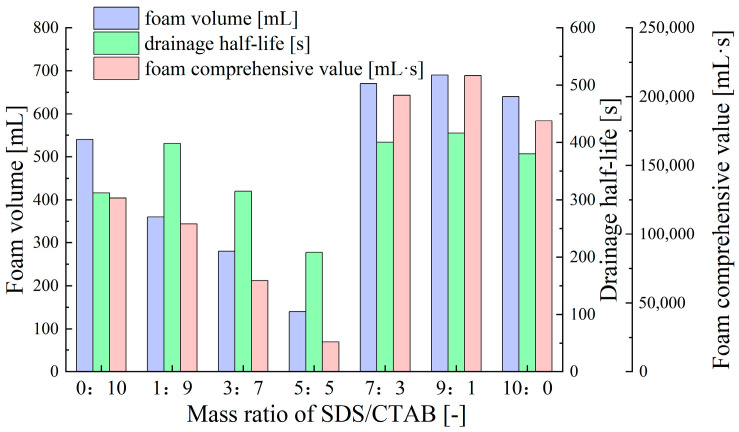
Effect of SDS/CTAB ratio on foam performance at 1% total surfactant concentration.

Figure 2 shows the foam volume, drainage half-life, and foam comprehensive value of foam formed by the compounded surfactant with different mass ratios of SDS (anionic surfactant) and CTAB (cationic surfactant). As shown in Figure 2, as the SDS ratio increases, the foam volume, foam half-life, and foam comprehensive value of the compounded surfactant foam first decrease and then increase. The maximum values are reached when the mass ratio of anionic to cationic surfactants is 9:1, demonstrating the best foaming performance and stability, which are superior to those of foam formed by single SDS and single CTAB surfactants. The main reason for this is that adding a small amount of cationic surfactant (CTAB) to the anionic surfactant (SDS) weakens the electrostatic repulsion between the head groups of the anionic surfactants, allowing the surfactant molecules in the adsorption layer to arrange more closely. At the same time, it retains some charge to form a double-layer structure, thereby enhancing the stability of the foam [48]. When the SDS to CTAB mass ratio is 5:5, the cationic and anionic surfactants tend to aggregate and sediment, resulting in the loss of surface activity and the poorest foam performance [49].

- Synergistic Effects of Anionic–Zwitterionic Surfactant Mixtures

**Figure 3:**
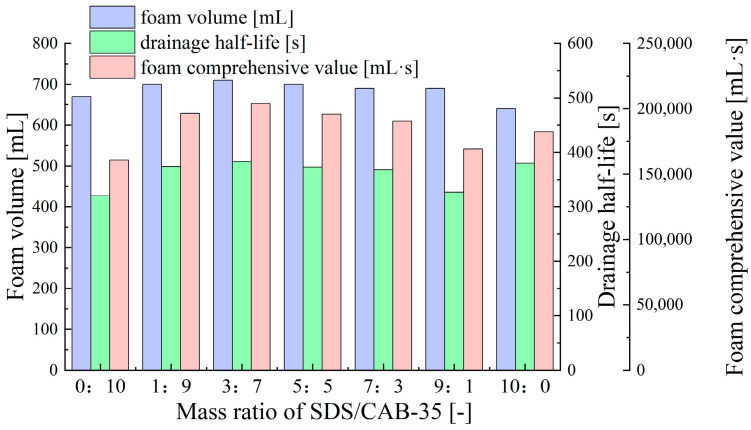
Effect of SDS/CAB-35 ratio on foam performance at 1% total surfactant concentration.

As presented in Figure 3, compared to the anionic–cationic compounded surfactant, the anionic–zwitterionic compounded surfactant (SDS-CAB-35) exhibits higher foam volume, and the fluctuations in drainage half-life and foam comprehensive value are significantly reduced with the mass ratio of SDS/CAB-35. When the mass ratio of SDS to CAB-35 is 3:7, the foam formed by the compounded surfactant reaches optimal foam volume, half-life, and foam comprehensive value, outperforming the foam formed by single SDS and CAB-35 surfactants. This phenomenon is attributed to the charge compensation effect of the zwitterionic surfactant CAB-35, which weakens the electrostatic repulsion of the anionic surfactant SDS. At the same time, the synergistic arrangement of the hydrophobic chains and polar head groups between molecules enhances the strength of the interfacial film, thereby improving foam comprehensive performance [50].

- Synergistic Effects of Anionic–Nonionic Surfactant Mixtures

**Figure 4:**
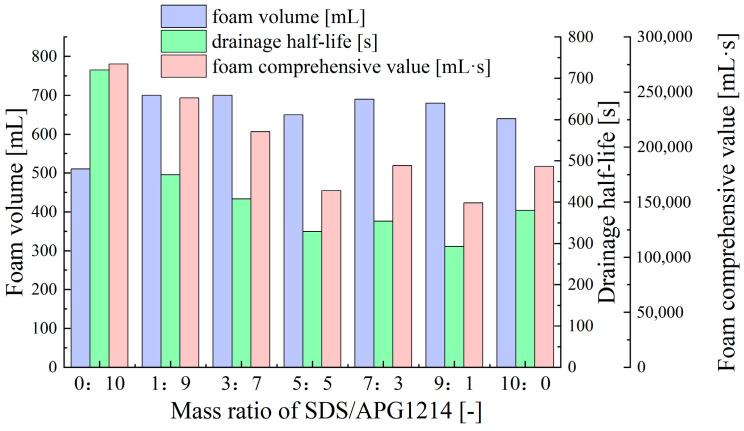
Effect of SDS/APG1214 ratio on foam performance at 1% total surfactant concentration.

As illustrated in Figure 4, as the APG1214 (nonionic surfactant) content increases, the drainage half-life and foam comprehensive value of the SDS-APG1214 compounded surfactant foam significantly improve, while the foam volume remains relatively stable. The optimal foam comprehensive performance is achieved when the SDS/APG1214 mass ratio is 1:9. This is closely related to the properties of nonionic surfactants; nonionic surfactants can increase the viscosity of the solution, particularly near the gas–liquid interface. This results in a slower drainage rate of the foam liquid film, significantly increasing the foam half-life and enhancing foam stability [51].

- Synergistic Effects of Anionic–Anionic Surfactant Mixtures

**Figure 5:**
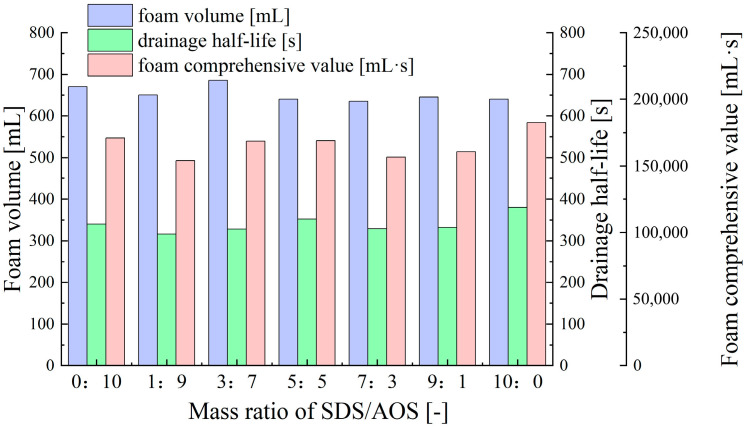
Effect of SDS/AOS ratio on foam performance at 1% total surfactant concentration.

Compared to the compounded surfactant discussed above, the foam volume, drainage half-life, and foam comprehensive value of the SDS-AOS compounded surfactant foam remain relatively stable at different SDS/AOS mass ratios (Figure 5). This indicates that change in the ratio of anionic surfactants has minimal impact on foam performance. As anionic surfactants, both SDS and AOS contain long carbon chains (hydrophobic part) and negatively charged sulfonate head groups (hydrophilic part) in their molecular structures, which are key factors in stabilizing the foam [52]. However, due to the similar molecular structures of SDS and AOS, their mechanisms of action during foam generation are also similar, resulting in similar interfacial behaviors.

In summary, through comparing various compounded surfactant with different mass ratios, the four compounded surfactants with optimal static foam comprehensive properties are found: the SDS-CTAB compounded surfactant with a mass ratio of 7:3, the SDS-CAB-35 compounded surfactant with a mass ratio of 3:7, the SDS-APG1214 compounded surfactant with a mass ratio of 1:9, and the SDS-AOS compounded surfactant with a mass ratio of 5:5.

#### 2.1.3. Determination of the Newly Developed Compounded Surfactant Formulation

The performance of foam in porous media is influenced by various factors, such as pore size, shape, and distribution, which means that the static performance of foam cannot fully reflect its dynamic behavior in porous media. In this section, the resistance coefficients of foam formed by the four compounded surfactants mentioned above are measured to evaluate the plugging performance of various foams in porous media [53].

**Figure 6:**
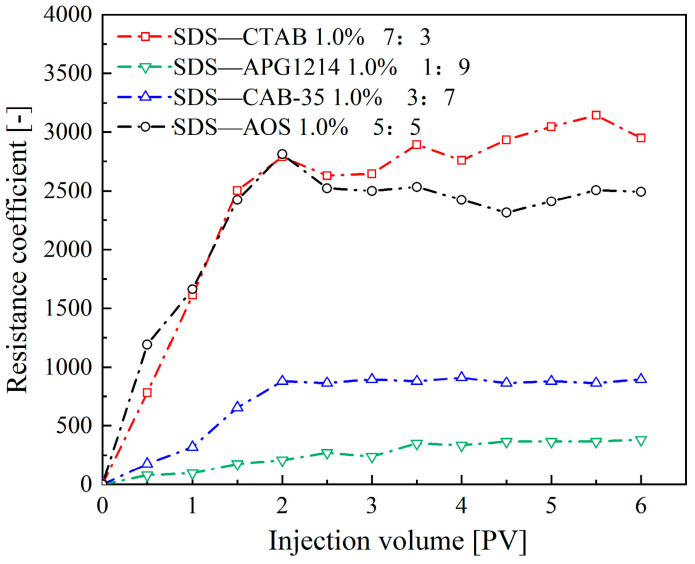
Resistance coefficients for various types of compounded surfactant foam.

As shown in Figure 6, as more foam is injected, the resistance coefficients of foam formed by the four compounded surfactant formulations first gradually increase and then reach a dynamic steady state at approximately 2 PV of foam injection. Overall, the SDS-CTAB compounded surfactant foam exhibits the highest resistance coefficient (approximately 2950), indicating the best plugging performance, followed by SDS-AOS, SDS-CAB-35, and SDS-APG1214 in descending order of performance.

Experimental results indicate that, among all tested compounded surfactants, the foam formed by the combination of sodium dodecyl sulfate (SDS) and cetyltrimethylammonium bromide (CTAB) exhibits the highest resistance coefficient in porous media. Therefore, we further investigate the plugging performance of SDS-CTAB compounded surfactant foam with different mass ratios to determine the optimal mass ratio.

**Figure 7:**
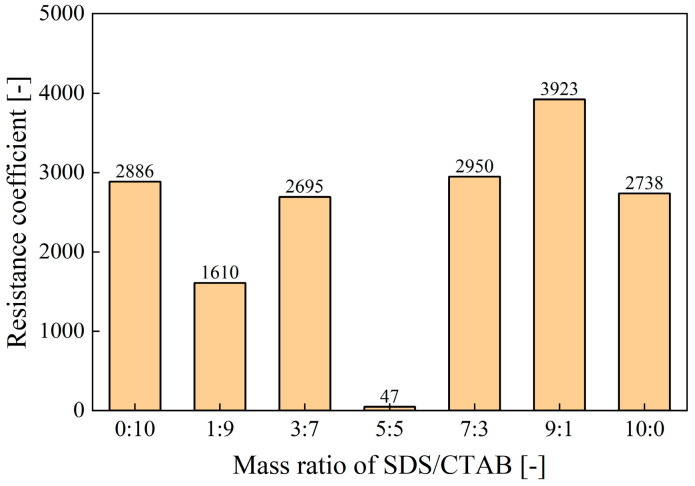
Effect of SDS/CTAB ratio on resistance coefficient at 1% total surfactant concentration.

As presented in Figure 7, at a surfactant concentration of 1%, the foam formed by the compounded surfactant with a mass ratio of 9:1 (SDS/CTAB) shows the highest resistance coefficient (3923), significantly higher than that of other mass ratios. This indicates that the foam achieves optimal plugging performance at this specific mass ratio.

Subsequently, the resistance coefficients of foams of varying surfactant concentrations, under the same compounded ratio, are analyzed, leading to the final determination of the newly compounded surfactant formulation.

**Figure 8:**
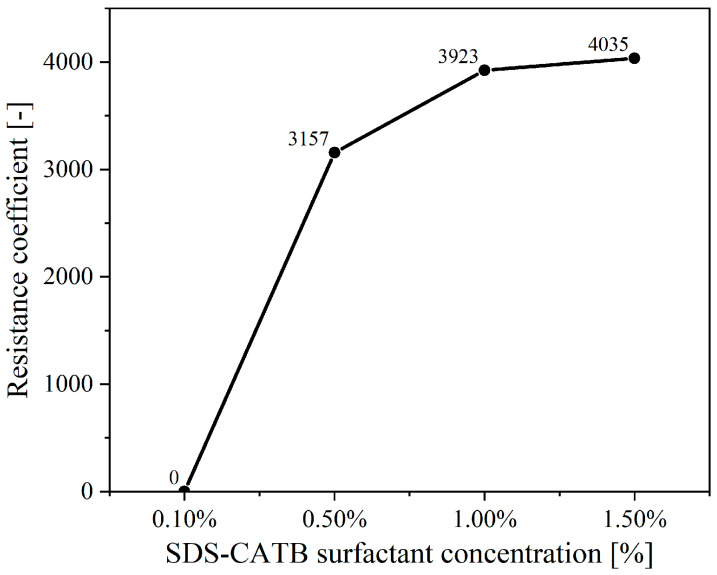
Effect of total surfactant concentration on resistance coefficient.

Accordingly, the SDS-CTAB compounded surfactant with a 9:1 mass ratio and a total surfactant concentration of 1.5% is identified as the newly developed compounded surfactant formulation in this study, due to its excellent static foam property [44] and effective plugging performance in porous media (Figure 8).

### 2.2. Effect of Newly Developed Surfactant Foam in Restraining Piping

In order to illustrate the effectiveness of the newly developed surfactant (SDS-CTAB) foam for restraining piping development, a series of experiments are conducted, including the traditional piping experiment, which is consistent with the gradient range reported by Van Beek et al. for uniform sands [54]; the SDS surfactant foam restraining piping experiment; and the SDS-CTAB surfactant foam restraining piping experiment. The piping initiation hydraulic gradient, the critical hydraulic gradient for piping, the cumulative sand loss, the water flow rate at the outlet, and the length of the piping channel are adopted to evaluate the effectiveness of foam in restraining piping development. The results are discussed in detail as follows.

#### 2.2.1. Enhancing the Initiation and Critical Hydraulic Gradient of Piping

**Figure 9:**
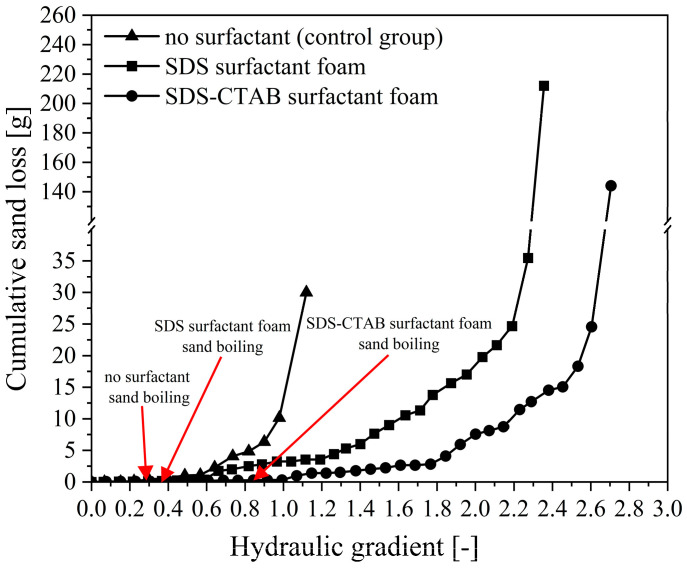
Cumulative amount of sand loss with increasing hydraulic gradient.

The point of sand boil at the outlet indicates the initiation of piping. As shown in Figure 9, in the case without surfactant injection, the piping initiation hydraulic gradient is approximately 0.29; when a certain amount of SDS foam is injected, it increases to about 0.35. In contrast, in the case of SDS-CTAB foam injection, the piping initiation hydraulic gradient increases significantly to approximately 0.85.

Figure 9 shows the cumulative amount of sand loss with increasing hydraulic gradient. Clearly, both types of foam are effective in delaying sand particle migration, with the newly developed SDS-CTAB surfactant foam exhibiting a more pronounced effect. Without surfactant injection, the cumulative amount of sand loss increases sharply once the hydraulic gradient reaches 0.6. With the injection of SDS foam, the cumulative amount of sand loss begins to gradually increase when the hydraulic gradient reaches 1.2. In contrast, when the SDS-CTAB foam is injected, a noticeable increase in the cumulative amount of sand boiling is not observed until the hydraulic gradient exceeds 1.8.

The hydraulic gradient corresponding to the end point of the curve in Figure 9 represents the critical hydraulic gradient for piping. The newly developed SDS-CTAB surfactant foam can significantly increase the critical hydraulic gradient of piping. As the hydraulic gradient rises to the critical hydraulic gradient, intense sand boiling occurs at the outlet, and the piping channel extends to the inlet chamber quickly. The critical hydraulic gradient for piping in the case without surfactant injection is approximately 1.12. With the injection of SDS surfactant foam, this value increases to 2.35, representing a 109% increase compared to the no surfactant case. When the newly developed SDS-CTAB compounded surfactant foam is injected, the critical hydraulic gradient rises further, to about 2.70, indicating a 141% increase relative to the no surfactant case and a 15% increase compared to the SDS foam. This clearly demonstrates the superior effectiveness of the compounded surfactant foam in enhancing soil resistance to piping.

#### 2.2.2. Restraining Piping Channel Extension

**Figure 10:**
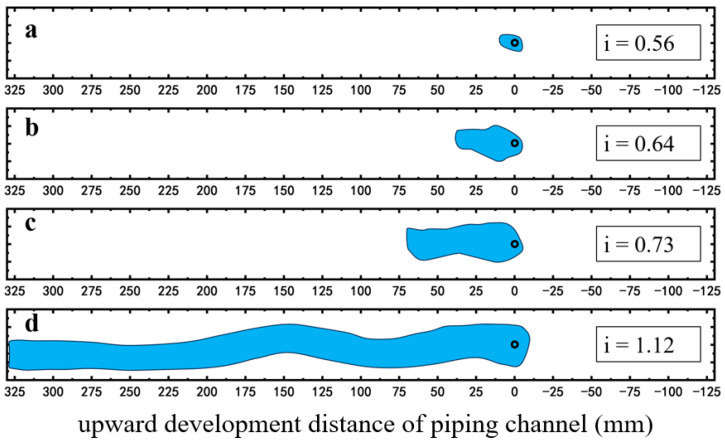
Evolution process of the piping channel with increasing hydraulic gradient i. (**a**) i = 0.56; (**b**) i = 0.64; (**c**) i = 0.73; (**d**) i = 1.12.

As presented in Figure 10, in the case of no surfactant injection, at low hydraulic gradients, no apparent piping channel is observed [55]. As the hydraulic gradient increases to 0.56, a distinct piping area appeared on the top of the sample (Figure 10a). Under a constant hydraulic gradient, the amount of sand loss decreases gradually until it ceases, and the development of the piping area stops accordingly. As the hydraulic gradient rises to 0.64, lateral expansion of the piping channel occurs near the outlet (Figure 10b). The piping channel extends continuously as the hydraulic gradient increases. At a hydraulic gradient of 0.73, the piping area extends about 7 cm (Figure 10c). When the hydraulic gradient reaches 1.12, the piping channel penetrates to the inlet chamber along the top surface (Figure 10d), accompanied by a large amount of sand loss.

**Figure 11:**
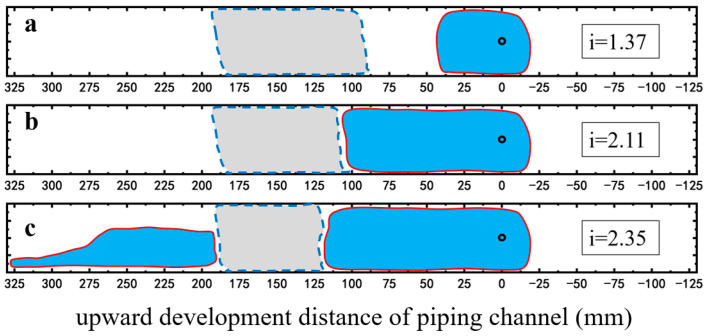
Development of piping channel in the case of SDS surfactant foam injection. (**a**) i = 1.37; (**b**) i = 2.11; (**c**) i = 2.35. The red lines represent the boundary of the piping channel, and the dashed lines represent the boundary of the foam area observed from the top surface.

**Figure 12:**
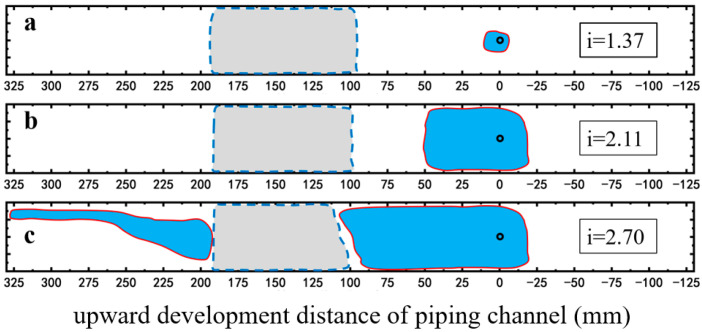
Development of piping channel in the case of SDS-CTAB surfactant foam injection. (**a**) i = 1.37; (**b**) i = 2.11; (**c**) i = 2.35. The red lines represent the boundary of the piping channel, and the dashed lines represent the boundary of the foam area observed from the top surface.

Foam significantly alters the development pattern of the piping channel. On the right side of the foam area, the piping area changes from a channelized form, observed under the no surfactant condition, to an integrated form (Figure 11 and Figure 12). On the left side of the foam area, upon reaching the critical hydraulic gradient for piping, a channelized piping area develops near the inlet chamber, while the piping area adjacent to the left boundary of the foam area exhibits a semi-integrated form with a width approximately half that of the piping area on the right side (Figure 11c and Figure 12c). In both cases, the foam area remained unbreeched, indicating the foam’s barrier effect against piping development. In the case of SDS-CTAB surfactant foam injection, an obvious piping channel is not observed until the hydraulic gradient reaches 1.37 (Figure 12a), whereas at the same hydraulic gradient, the SDS surfactant foam experiment has already developed a piping channel approximately 4.5 cm in length (Figure 11a). When the hydraulic gradient increases to 2.11, the piping channel in the SDS surfactant foam case propagates to the edge of the foam area, approximately 10 cm from the outlet (Figure 11b), while under the same hydraulic gradient of 2.11, the piping channel propagates only about 5 cm in the SDS-CTAB surfactant foam case at the same hydraulic gradient of 2.11 (Figure 12b). Therefore, the newly developed SDS-CTAB surfactant foam exhibits more pronounced inhibitory effect on the development of the piping channel compared to the single-component (SDS) surfactant foam.

#### 2.2.3. Reducing Flow Rate at Outlet

**Figure 13:**
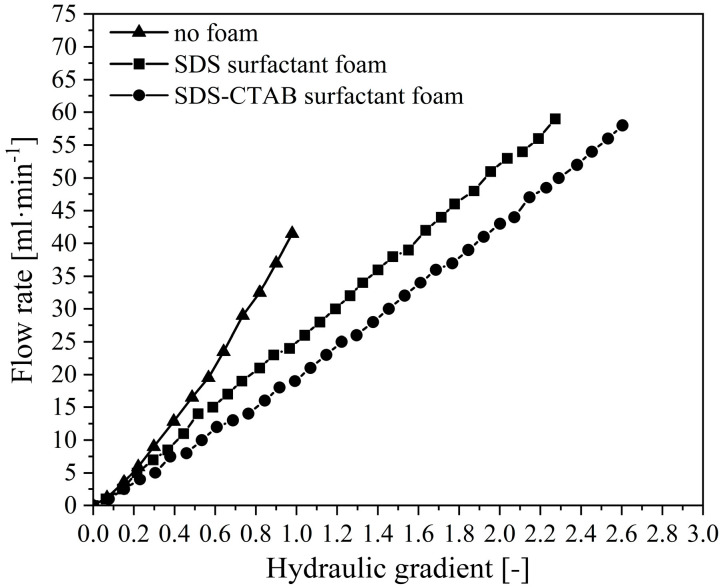
Flow rate at various hydraulic gradients.

During the experiments, the water flow rate at the outlet under different hydraulic gradients is measured. As presented in Figure 13, under the no surfactant condition, the flow rate increases nonlinearly with the hydraulic gradient, which is in good accordance with previous studies. In contrast, the flow rate curves under foam-injection conditions exhibit a more linear trend. The exitance of foam clearly reduces the water flow rate; at a certain hydraulic gradient, the cases with foam injection present lower flow rates, especially the cases with the newly developed SDS-CTAB surfactant foam. This is primarily due to the formation of a uniform and continuous foam structure within the pores of the sand body. This structure effectively inhibits the loosening and migration of soil particles, thereby maintaining the relative stability of the internal pore structure. As a result, the abrupt increase in flow rate caused by sudden seepage failure, observed under the no surfactant condition, is not observed.

### 2.3. Mechanisms of Compounded Surfactant Foam Enhancing Capability in Restraining Piping Development

The experimental results demonstrate that, compared to single-component surfactant foam, the newly developed compounded surfactant foam significantly enhances both the initiation and critical hydraulic gradients for piping, inhibits sand particle migration, suppresses the development of piping channel, and effectively reduces the water flow rate at the outlet. These findings indicate the superior capability of the newly developed compounded surfactant foam in restraining piping. In this section, we further investigate the mechanisms by which the compounded surfactant foam enhances the capability in restraining piping. 

Section 2.3 conducts internal pressure cloud map analysis, interface effect analysis, and grayscale image analysis. These methods are employed to evaluate foam strength, stability, and anti-scour capacity, providing insights into the mechanism by which the compounded foam improves the soil’s resistance to seepage failure.

#### 2.3.1. Enhancement of Foam Strength

**Figure 14:**
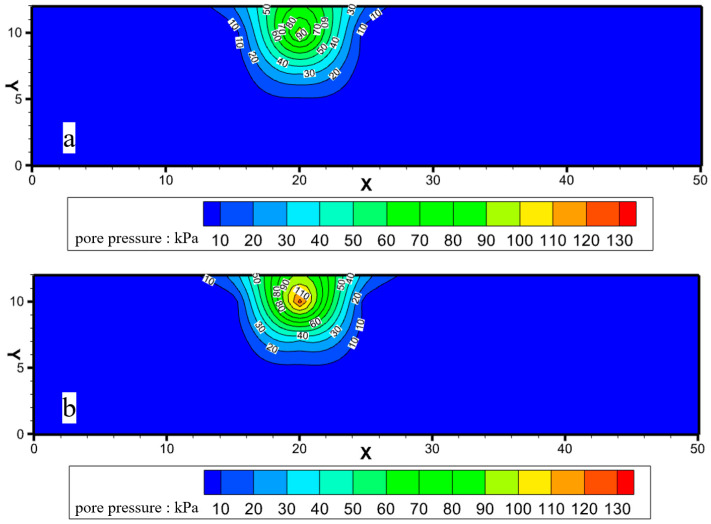
Initial pore pressure contour map: (**a**) injection of SDS surfactant foam; (**b**) injection of SDS-CTAB surfactant foam.

When SDS surfactant foam and SDS-CTAB surfactant foam were injected into the model, the injection pressures were basically the same (0.103 MPa for SDS surfactant foam and 0.109 MPa for SDS-CTAB surfactant foam) and under the same injection time conditions (45 min), the diffusion ranges of the two types of foam fluids were basically the same. However, the injection point pressure (*P*_3_) of SDS surfactant foam was 94 kPa, while the injection point pressure (*P*_3_) of SDS-CTAB surfactant foam reached 137 kPa, and the internal pressure increased by more than 46%, and the pressure difference between adjacent positions (Δ*P*_2-3_ and Δ*P*_4-3_) was larger, indicating that the strength of SDS-CTAB foam fluid in this area was higher (Figure 14), which greatly improved the fluid flow resistance in the foam fluid storage area. This is consistent with the plugging performance evaluation experiments.

#### 2.3.2. Enhancement of Foam Stability

According to the foam static-property characterization experimental results, the foam volume and drainage half-life of the SDS-CTAB compounded surfactant foam are both superior to those of the single-component surfactant foam. In the foam plugging performance test, the resistance coefficient of the SDS-CTAB compounded surfactant foam is also significantly higher than that of the single-component surfactant foam. These results indicate that the SDS-CTAB compounded surfactant, due to its synergistic effects, can effectively enhance the foam stability. The following section analyzes its synergistic effect mechanism [56].

Sodium dodecyl sulfate (SDS) is an anionic surfactant whose molecular structure contains a negatively charged sulfate group (-SO_4_) and a hydrophobic alkyl group (C_12_H_25_) [57]. It reduces the surface tension of water, facilitating foam generation. Cetyltrimethylammonium bromide (CTAB) is a cationic surfactant composed of a quaternary ammonium salt group (-N^+^(CH_3_)_3_) and a hydrophobic long-chain alkyl group (C_16_H_33_) [58].

- Electrostatic Interaction

**Figure 15:**
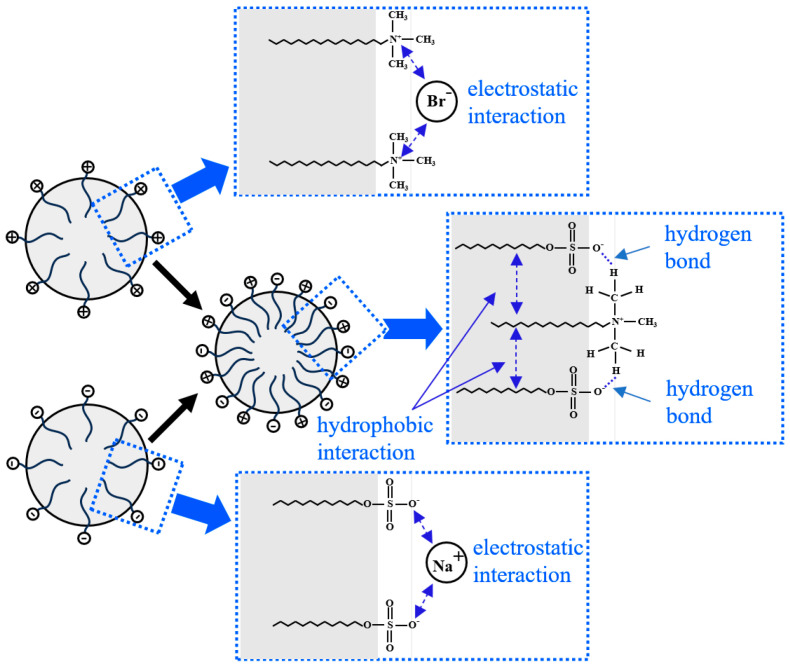
Schematic interfacial structure of binary anionic–cationic surfactant mixture (SDS-CTAB).

As shown in Figure 15, there is a natural electrostatic attraction between the positively charged portion of CTAB (the nitrogen ion) and the negatively charged portion of SDS (the sulfate group). This attraction between opposite charges helps stabilize the mixture of the two surfactants in aqueous solution. The bromide ion (Br^−^) in CTAB acts as a counterion and is partially attracted to the positively charged portion of the CTAB molecule. These ions help neutralize the positive charge of CTAB, reducing the electrostatic repulsion between CTAB molecules and making it easier for CTAB molecules to approach and interact with SDS molecules. Similarly, as an anionic surfactant, SDS contains negatively charged sulfate groups in its molecular structure. Sodium ions interact with these negatively charged groups, partially neutralizing them. By neutralizing the negative charge on the SDS molecules, sodium ions help reduce the electrostatic repulsion between SDS molecules, promoting aggregation. These electrostatic interactions effectively enhance the stability of the compounded system.

- Hydrophobic Interaction

SDS and CTAB, through the close packing of their hydrophobic tails, significantly enhance hydrophobic interactions. This strong hydrophobic effect helps reduce the gas diffusion rate within the bubbles, inhibiting the dissipation of the liquid film and enhancing the overall stability of the foam. Additionally, the stable liquid film effectively lowers the interfacial tension between water and air, facilitating foam generation (Figure 15).

- Hydrogen Bond

When the salt ion concentration in the solution is low, SDS and CTAB molecules form hydrogen bonds between the sulfate group of SDS and the N-methyl group of CTAB, creating C-H···O hydrogen bonds. The formation of these hydrogen bonds results in short-range attractive interactions [59]. The short-range attraction at the molecular level caused by hydrogen bonds allows the surfactant molecules to arrange more closely at the interface, restricting the mobility of the surfactant molecules and enhancing the elasticity of the surfactant absorption layer. This inhibits foam deformation and collapse, improving the overall stability of the foam (Figure 15).

#### 2.3.3. Enhancement of Scour Resistance of Foam

**Figure 16:**
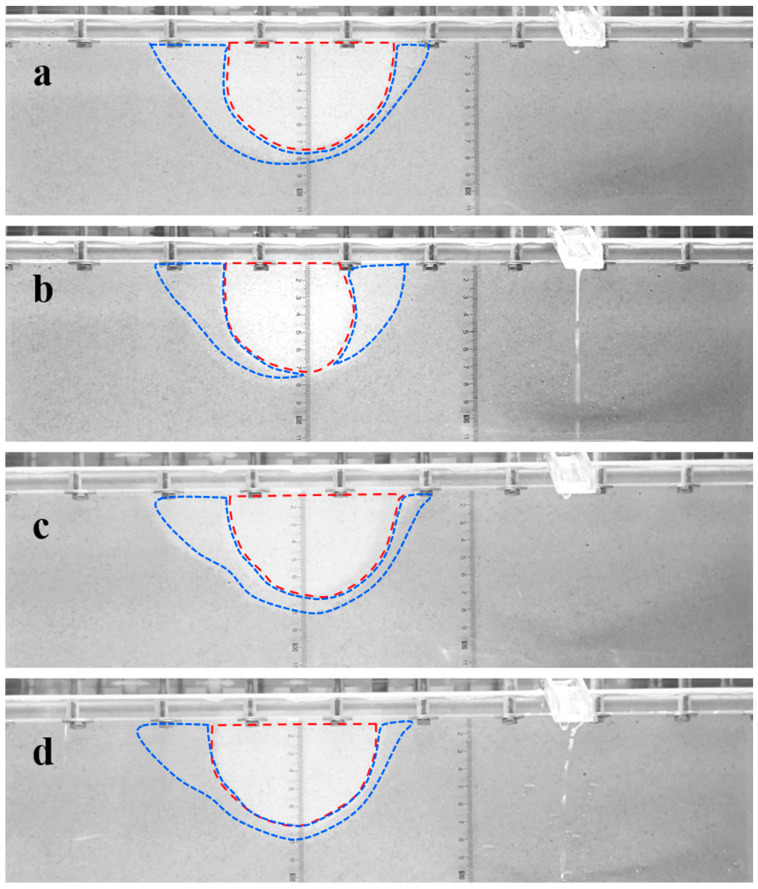
Side grayscale images of foam area: (**a**) initial state of SDS surfactant foam; (**b**) after 140 min of SDS surfactant foam restraining piping; (**c**) initial state of SDS-CTAB surfactant foam; (**d**) after 140 min of SDS-CTAB surfactant foam restraining piping. The red dashed lines represent the boundary of the strong foam zone, and the blue dashed lines represent the boundary of the weak foam zone.

The foam area can be identified in the grayscale images of the sand sample; the grayscale value of the foam area is higher than that of water-saturated area. The stronger the foam is, the higher the grayscale value. Figure 16a,b show the initial state of SDS surfactant foam, and the foam distribution after a 140-minute piping experiment, respectively. The red dashed line region, lighter in color (higher grayscale value), represents the strong foam zone, while the blue dashed line region, darker in color (lower grayscale value), represents the weak foam zone. During the experiment, factors such as water flow scouring and dilution can lead to the loss of surfactants, resulting in decreased foam strength. As shown in Figure 16b, after 140 min of scouring, the foam strength in the weak foam zone on the left side further decreased (color darkened), and the grayscale value of the right part of the originally strong foam zone significantly decreased (color darkened), transforming into a weak foam zone, resulting in significant reduction in strong-foam zone. The strong and weak foam zones of the SDS-CTAB surfactant foam are similar to those of the SDS surfactant foam at initial stage (Figure 16c). Similarly, after 140 min of scouring, the foam strength in the weak-foam zone further decreased. However, the strong foam zone remains almost unchanged (Figure 16d), indicating that the SDS-CTAB surfactant foam maintains higher strength and stability after water flow scouring and dilution. This suggests that the SDS-CTAB surfactant foam has greater scour resistance than the SDS surfactant foam under high hydraulic gradients and prolonged water flow scouring conditions, maintaining its capability for restraining piping.

## 3. Materials and Methods

### 3.1. Experimental Materials

In this study, distilled water and sodium chloride (NaCl) are utilized to prepare experimental water with a salinity of 236.63 mg/L, simulating the mineralization characteristics of the Yangtze River. Surfactant solutions of varying concentrations are prepared to serve as the liquid phase of foam. All surfactants listed in Table 1 were obtained from Tianjin Xienci Biochemical Technology Co., Ltd. and Shandong Yousuo Chemical Technology Co., Ltd., with their respective purities detailed in Table 1. High-purity nitrogen (99.999%), provided by Nanjing Ningwei Medical Oxygen Co., Ltd., Nanjing, China, is applied as the gas-phase of foam. The nitrogen and surfactant solution are injected into the porous media foam generator at a gas-liquid ratio of 0.7 (gas flow accounting for 70% of the total flow) and an apparent total flow rate of 2 mL/min to generate foam.

Environmental factors such as pH, salinity, and temperature can significantly affect the foam’s performance in porous media. In this study, all tests were conducted under controlled conditions at a temperature of 25 ± 5 °C and a neutral pH of approximately 7.0 ± 1, in order to minimize environmental interference. The influence of these factors will be considered in future work aimed at field-scale applications.

**Figure 17:**
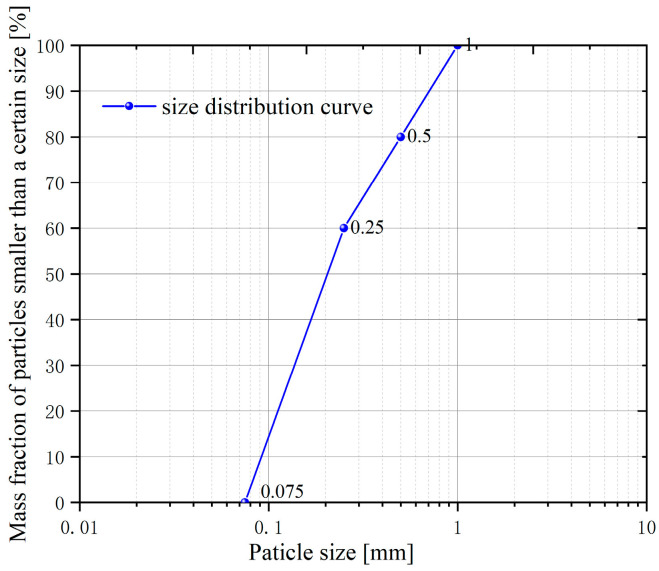
Sample particle size distribution.

Chinese ISO standard sand with particle sizes ranging from 0.075 mm to 1 mm are adopted to prepare the sand samples in the experiments of foam plugging performance evaluation and the ability of the foam to restrain piping development. The porosity of the sand sample is approximately 0.35, and its permeability is about 1.6 × 10^4^ mD. The particle size distribution is shown in Figure 17.

### 3.2. Experimental Apparatus

This study primarily conducts three types of experiments to evaluate the static performance, plugging performance, and application of foam in restraining seepage failure and development in dyke soil, as well as its control effect on piping and other seepage failures.

In this study, we evaluate the performance of foam and their efficacy in controlling piping through three experimental modules: (1) static-property characterization, (2) plugging capacity assessment, and (3) application of foam in restraining piping development.

#### 3.2.1. Apparatus for Foam Static-Property Characterization

**Figure 18:**
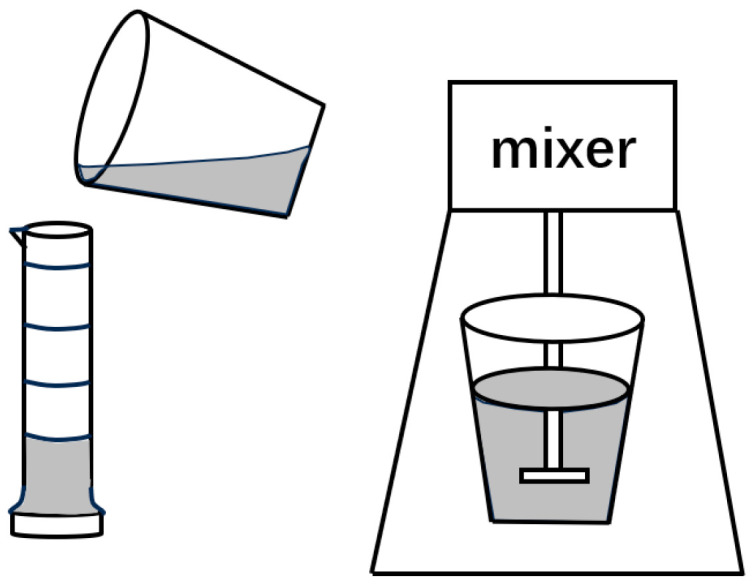
Schematic of Waring–Blender high-speed stirring method.

The static performance of foam is tested by applying the Waring–Blender high-speed stirring method [60]. The test device is HTDD-B12K frequency conversion high-speed stirrer produced by Qingdao Hengtaida Electromechanical Equipment Co., Ltd., with an idle speed range of 0-12,000 r/min, and a maximum stirring volume of 350 mL (Figure 18).

#### 3.2.2. Apparatus for Foam Plugging Capacity Assessment

**Figure 19:**
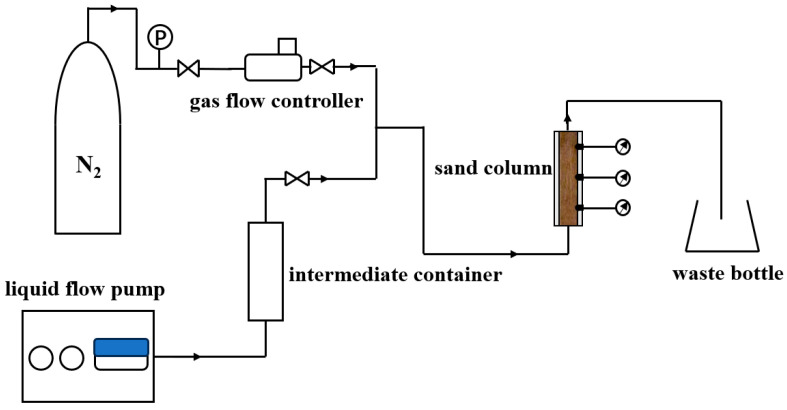
Schematic of foam plugging capacity assessment apparatus.

The foam plugging performance evaluation is conducted through a sand column model test device (Figure 19). The sand column model has a diameter of 4.6 cm and a length of 10.6 cm and is fabricated from transparent acrylic to facilitate real-time visualization of foam distribution within the sand column. The gas is injected into the sand column from the bottom via a mass-flow controller, while a liquid pump controls the injection rate of the surfactant solution, also from the bottom of the sand column. Foam is then generated in the sand and reaches a dynamic steady state.

#### 3.2.3. Apparatus for Foam Restraining Piping Experiment

**Figure 20:**
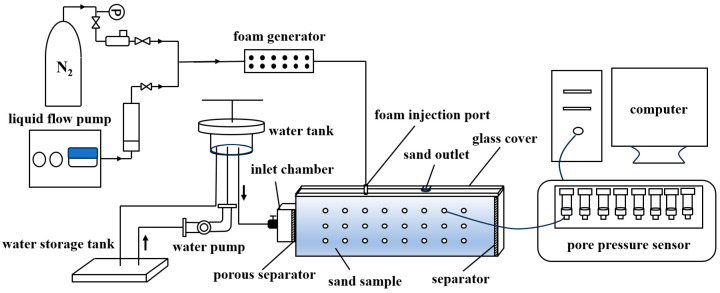
Schematic of foam restraining piping experimental apparatus.

In order to investigate the capability of foam to restrain piping development, a self-developed experimental system is employed to conduct the traditional piping experiment and the foam restraining piping experiment. The system comprises five core parts: the hydraulic head control system, the foam injection system, the sandbox model, the real-time pore pressure monitoring system, and the data acquisition system (Figure 20).

The hydraulic head control system: This system implements a variable-head device to precisely adjust the hydraulic head in the inlet chamber of the sandbox, enabling stepwise escalation of the hydraulic gradient for controlled experimental conditions.

The foam injection system: Surfactant solution and gas is first injected into a foam generator to produce foam; the flow rate of gas and liquid are controlled by a mass flow controller and a liquid pump, respectively. Steady-state foam is then injected into the sandbox through a preset injection port.

The sandbox model: To enable comprehensive experimental observation while minimizing soil disturbance from piezometers and other instruments, this study employs a small-scale transparent polymethyl methacrylate (PMMA) model with dimensions of 50 cm (L) × 4 cm (W) × 12 cm (H). The narrow width primarily affects the morphology of piping channels, but exhibits negligible effects on the overall development of piping [61]. The left side of the model serves as the inlet chamber, with a perforated partition plate placed between the sand sample and the inlet chamber to allow uniform water flow into the sample. Additionally, a geotextile filter is placed at the interface between the perforated partition plate and the sand sample to prevent sand particles from entering the inlet chamber. The top of the sandbox is sealed with a transparent polymethyl methacrylate plate, with a silicone pad placed between the cover and the sand sample. During the experiment, Vaseline is applied to the sidewalls of the model, and bolts are used to tightly secure the seal and prevent leakage. A 5 mm diameter water outlet is preset on the cover at a position of 35 cm from the inlet chamber to simulate the formation of a sand boil orifice of the overburden layer.

**Figure 21:**
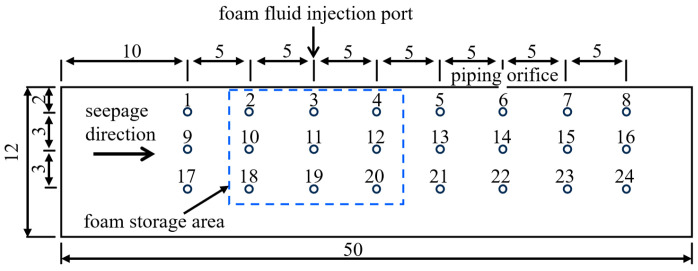
Schematic of pressure sensors arrangement (unit: cm).

The real-time pore pressure monitoring system: This system includes three rows of pressure measuring points installed on the side of the sandbox, each row containing eight points, to monitor pore pressure at different locations in real time, and assess the impact of foam on the seepage field. The foam injection port is inserted 2 mm into the sand sample, with the top row of measuring holes positioned 5 mm below the cover. To ensure accurate representation of pore pressure distribution under various experimental conditions, pressure sensors with different measurement ranges are employed. In the piping experiments without surfactant, 24 pressure sensors with a range of 0–20 kPa are used. In the experiments of foam restraining piping development, 15 pressure sensors with a range of 0–20 kPa and 9 with a range of 0–200 kPa are used. The 0–200 kPa sensors are symmetrically arranged around the foam injection port (Figure 21).

The data acquisition system: During the experiment, the expansion of the piping channel and the diffusion range of foam are recorded by a high-speed camera, while the flow rate and sand loss data are also collected. All related data are combined together to perform substantial analysis.

### 3.3. Experimental Method

The experiments of this study can be divided into two main phases. First, surfactants are selected through static property and plugging performance evaluation experiments, thereby the optimal compounded surfactant formulation is determined. Second, comparative experiments of piping and enhanced foam restraining the piping are performed to evaluate the effect and mechanisms of the newly developed foam controlling piping development. The specific experimental methods are described as follows.

#### 3.3.1. Foam Static-Property Characterization Experiment

In this study, the Waring–Blender method [62] is employed to evaluate the foamability and stability of single-component and compounded surfactants. First, 100 mL prepared surfactant solution is added to the mixing cup and stirred at 8000 r/min for 3 min to generate foam. Then, the generated foam and liquid are quickly transferred into a 1000 mL measuring cylinder and the foam volume *V*_0_ (mL) and drainage half-life *t*_1/2_ (s) are recorded. Each test was repeated three times under identical conditions, and the average values were used for analysis. Since the overall performance of foam is influenced by both foamability and stability, the foam comprehensive value (*FCI*) is adopted as the primary parameter for evaluating foam static property [63].

(1) $$\mathrm{FCI}=\frac{3}{4}\;\times \;V_{0}\;\times {\;t}_{1/2}.$$

In the equation, *FCI* denotes the foam comprehensive value (mL·s), *V*_0_ represents the foam volume (mL), and *t*_1/2_ is the drainage half-life (s).

#### 3.3.2. Foam Plugging Capacity Assessment Experiment

In this study, a sand column model is employed to conduct foam and water injection experiments. Foam and liquid are injected into the sand sample at the same flow rate of 2 mL/min and the pressure difference along the sand sample is monitored to calculate the resistance coefficient [53] of the foam, thereby evaluating its plugging capability. In the foam experiment, 1.5 PV surfactant solution is injected beforehand to fully saturate the sample and facilitate surfactant adsorption. The pressure difference across the sample for steady-state foam is taken for calculation. The resistance coefficient is defined as follows:

(2) $$R_{F}=\frac{\Delta P_{\mathrm{foam}}}{\Delta P_{w}}{|}_{Q=\mathrm{const}}$$

In the equation, *R_F_* is the resistance coefficient (dimensionless), $\Delta P_{\mathrm{foam}}$ is the pressure difference measured across the sand column during foam injection (Pa), and $\Delta P_{w}$ is the water-driven pressure difference under the same total flow rate *Q* (Pa).

#### 3.3.3. Experiment of Foam Restraining the Piping

This series of experiments aim to compare the development process of piping under three conditions: no surfactant injection, single-component surfactant foam injection, and newly developed surfactant foam injection. The comparison is based on data including water flow rate and sand loss at the outlet, pore pressure distribution, and visualized piping channel at different water head. In the piping experiment, the upstream water head is gradually increased to raise the hydraulic gradient, with each increment being approximately 5 cm. Once seepage stabilizes, the next water head is applied. Seepage is considered stable when the water flow rate at the outlet remains stable after multiple measurements, with no significant sand particle movement and the water remaining clear. The water flow rate and the amount of sand loss are recorded at each water head, while changes in the piping area at the top of the sand sample and the distribution of foam are also monitored.

In the experiments involving foam injection, foam is first generated through a foam generator, with a gas-to-liquid ratio of 0.7, and a superficial flow rate of 2 mL/min. Once the pre-generated foam reaches dynamic steady-state, it is then injected into the sand pack through a foam injection port located 15 cm upstream of the outlet, with the foam outlet extending 2 cm into the sand sample. The injection duration is 45 min. The diffusion of foam within the sand sample and the corresponding changes in pore pressure are observed and recorded. After the completing foam injection, the piping experiment is performed.

## 4. Conclusions

In this study, we address the specific requirements for employing foam in emergency mitigation of piping in dikes. An anion–cation compounded surfactant foam is developed by utilizing the synergistic effects of cationic-anionic surfactant mixtures. The SDS-CTAB compounded surfactant with a 9:1 mass ratio and a total surfactant concentration of 1.5% is identified as the optimum surfactant formulation in this study, due to its excellent static foam property and effective plugging performance in porous media. The anion–cation compounded surfactant foam enhances the initiation and critical hydraulic gradient of piping, inhibits piping channel extension, and reduces the water flow rate at the outlet, thereby presenting excellent performance in restraining piping development. Compared to the single-component foam, the anion–cation compounded surfactant foam is stronger and more stable, due to the following reasons: the electrostatic interaction between the positively charged portion of CTAB (the nitrogen ion) and the negatively charged portion of SDS (the sulfate group) and the hydrophobic interaction and the hydrogen bond. The scour resistance of foam is also improved, which means that the newly developed foam can better maintain its capability for restraining piping under high hydraulic gradients and prolonged water flow scouring conditions.

## Figures and Tables

**Table 1:** Single-component surfactant foam performance.

Surfactant Types	Surfactants	Concentration	*V*_0_	*t*_1/2_	*FCI*
Anionic surfactant	Sodium alpha olefin sulfonate (AOS, 92% purity, Tianjin Xienci Biochemical Technology Co., Ltd., Tianjin, China)	0.1%	615	329	151751
		0.3%	645	353	170763
		0.5%	630	369	174352
		0.7%	650	344	167700
		0.9%	665	326	162592
	Sodium dodecyl sulfate (SDS, 99% purity, Tianjin Xienci Biochemical Technology Co., Ltd.)	0.1%	600	330	148500
		0.3%	630	363	171517
		0.5%	640	369	177120
		0.7%	635	354	168592
		0.9%	630	375	177187
	Disodium sulfosuccinate monoester (MES-30, 30% active content, Shandong Yousuo Chemical Technology Co., Ltd., Linyi, China)	0.1%	530	254	100965
		0.3%	600	310	139500
		0.5%	620	280	130200
		0.7%	630	315	148837
		0.9%	650	272	132600
	Sodium fatty alcohol ether sulfate (AES, 70% active content, Shandong Yousuo Chemical Technology Co., Ltd.)	0.1%	540	248	100440
		0.3%	610	229	104767
		0.5%	630	265	125212
		0.7%	620	238	110670
		0.9%	620	254	118110
Zwitterionic surfactant	Cocoamidopropyl betaine (CAB-35, 35% active content, Tianjin Xienci Biochemical Technology Co., Ltd.)	0.1%	590	273	120802
		0.3%	650	263	128212
		0.5%	650	290	141375
		0.7%	660	271	134145
		0.9%	660	318	157410
	Lauryl propyl betaine (LAB-35, 35% active content, Tianjin Xienci Biochemical Technology Co., Ltd.)	0.1%	430	190	61275
		0.3%	570	268	114570
		0.5%	650	290	141375
		0.7%	640	275	132000
		0.9%	630	282	133245
	Lauramidopropyl hydroxy sulfobetaine (LHSB, 35% active content, Shandong Yousuo Chemical Technology Co., Ltd.)	0.1%	470	182	64155
		0.3%	600	230	103500
		0.5%	600	257	115650
		0.7%	600	276	124200
		0.9%	610	270	123525
	Dodecyl dimethyl amine oxide (OB-2, 98% purity, Tianjin Xienci Biochemical Technology Co., Ltd.)	0.1%	620	271	126015
		0.3%	640	234	112320
		0.5%	630	190	89775
		0.7%	630	194	91665
		0.9%	620	158	73470
Nonionic surfactant	Coconut diethanolamide (CDEA, 70% active content, Shandong Yousuo Chemical Technology Co., Ltd.)	0.1%	120	310	27900
		0.3%	590	307	135847
		0.5%	670	325	163312
		0.7%	600	381	171450
		0.9%	330	1052	260370
	Alkyl glycoside (APG1214, 50% active content, Shandong Yousuo Chemical Technology Co., Ltd.)	0.1%	380	266	75810
		0.3%	510	392	149940
		0.5%	550	427	176137
		0.7%	560	561	235620
		0.9%	530	668	265530
	Polysorbate 80 (Tween 80, 99% purity, Shandong Yousuo Chemical Technology Co., Ltd.)	0.1%	80	275	16500
		0.3%	120	376	33840
		0.5%	350	147	38587
		0.7%	400	195	58500
		0.9%	450	209	70537
	Primary alcohol ethoxylate (AEO-9, 99% purity, Shandong Yousuo Chemical Technology Co., Ltd.)	0.1%	300	129	29025
		0.3%	470	195	68737
		0.5%	520	224	87360
		0.7%	550	243	100237
		0.9%	590	236	104430
Cationic surfactant	Cetyltrimethylammonium bromide (CTAB, 98% purity, Tianjin Xienci Biochemical Technology Co., Ltd.)	0.1%	475	290	103312
		0.3%	470	271	95527
		0.5%	515	297	114716
		0.7%	500	299	112125
		0.9%	550	300	123750
	Dodecyl trimethyl ammonium chloride (DTAC, 30% active content, Shandong Yousuo Chemical Technology Co., Ltd.)	0.1%	420	201	63315
		0.3%	475	267	95118
		0.5%	620	234	108810
		0.7%	600	229	103050
		0.9%	600	224	100800

## Data Availability

The original contributions presented in this study are included in the article. Further inquiries can be directed to the corresponding author.
